# Anaerobic Bacteria as a Cause of Mycotic Aneurysm of the Aorta: Microbiology and Antimicrobial Therapy

**DOI:** 10.2174/157340309787048095

**Published:** 2009-01

**Authors:** Itzhak Brook

**Affiliations:** Departments of Pediatrics and Medicine, Georgetown University School of Medicine, Washington DC, USA

**Keywords:** Anaerobic bacteria, mycotic aneurysm, aorta, *Bacteroides fragilis*, *Clostridium spp.*, *Propionibacterium acnes.*

## Abstract

This review summarizes the microbiology, and antimicrobial management of mycotic aneurysm of the aorta (MAA) due to anaerobic bacteria. Anaerobic bacteria are an uncommon but important cause of MAA. Most cases of anaerobic MAA are caused anaerobic gram-negative bacilli (mostly *B. fragilis *group), *Clostridium * spp. (mostly *Clostridium septicum*, *and Propionobacterium * spp. (mostly *P. acnes*)*. *Clostridial infection is frequently associated with gastrointestinal or hematologic malignancy. A review of all the reported cases is presented. Treatment of MAA involving anaerobic bacteria includes the use of antimicrobial effective against these organisms.

## INTRODUCTION

Infections due to anaerobic bacteria are common, and can be serious and life-threatening. The recent increased in the recovery of these organisms in all infectious sites [1] including bacteremia [2] has led to greater appreciation of the role anaerobes play in infections at all body sites, including mycotic aneurysm of the aorta (MAA). 

As the main components of the normal human skin and mucous membranes bacterial flora, anaerobes are a common cause of endogenous bacterial infections. Because of their fastidiousness, they are difficult to isolate from infected sites, and are often overlooked. Their exact frequency is hard to ascertain because of the inconsistent utilization of adequate methods for their isolation and identification. The lack of directing adequate antimicrobial therapy against these organisms may lead to clinical failures, complication and increase mortality. Their isolation requires appropriate methods of collection transportation and cultivation of specimens. Treatment of anaerobic infection is complicated by their polymicrobial nature, and by the slow growth and growing resistance to antimicrobials of anaerobic bacteria [3].

MAA is a life-threatening condition with significant morbidity and mortality. *Staphylococcus* and *Salmonella* spp. are the two most commonly cultured organisms in mycotic aneurysms. However, improved bacteriologic techniques have led to the detection of anaerobic bacteria (mostly *Bacteroides*, and *Clostridium* spp.) in MAA [4]. This review describes the microbiology and antimicrobial management of MAA due to anaerobic bacteria.

## MICROBIOLOGY

The study by Brook and Frazier [4] illustrated the diversity of anaerobic bacteria recovered from patients with MAA. The study was conducted between 1987 and 1992 and presented 8 cases with MAA who had aerobic and anaerobic cultures. Eleven organisms, 6 aerobic and facultative and 5 anaerobic, were isolated. Aerobic organisms only were isolated in 4 cases, anaerobic organisms only in 3, and mixed aerobic and anaerobic bacteria in one. Polymicrobial infection was present in three. The isolated aerobic bacteria were *Staphylococcus aureus* (2 isolates), and *Staphylococcus epidermidis, Escherichia coli, Klebsiella pneumoniae*, and *Salmonella enteritidis* (one each). The recovered anaerobes were *Peptostreptococcus* spp. (2), and *Bacteroides fragilis, Propionibacterium acnes,* and *Clostridium perfingens* (one each). Organisms similar to the one recovered from the MAA were isolated from the blood of 4 patients. These include one isolate each of *S. aureus*, *E. coli*, *B. fragilis*, and *C. perfringens*.

Several case reports of MAA due to anaerobic bacteria (mainly *B. fragilis* and *Clostridium septicum*) were published.

### *Bacteroides fragilis* Infections

Sheehan [5] presented a patient with aortitis due to *Bacteroides* spp. that developed MAA, which ruptured following translumbar aortography. Reddy *et al.* [6] reported a patient with a 7 cm saccular MAA due to *B. fragilis* that emerged in the suprarenal abdominal area.

Suddleson *et al.* [7] presented a case of mycotic suprarenal MAA caused by *B. fragilis*. The patient was treated only with continuous antibiotic therapy and had a complete recovery. Taylor *et al.* [8] described a patient with infected abdominal aortoiliac mycotic aneurysm caused by *Bacteroides* spp. The patient was treated with appropriate antibiotics and a single operative procedure consisting of preliminary extraanatomic bypass followed by complete aneurysm excision and posterior drainage of the retroperitoneum. Jewkes and Black [9] reported a case with MAA caused by a Gram-negative anaerobic organism. The MAA was in the abdominal area and was a consequence of an abscess of the appendix. 

Reddy *et al.* [10] performed a 30-year retrospective review and identified 13 patients treated for infected aneurysms of the abdominal aorta or iliac arteries. Four (31%) of the patients died within 30 days of operation, three of whom of aneurysmal rupture. *Salmonella* spp., *B. fragilis, S. aureus* and *Pseudomonas aeruginosa* accounted for all deaths, ruptures, and suprarenal aneurysm infections which occurred in 10 (77%) of the patients.

Hsu *et al.* [11] performed a retrospective review of all 19 patients with MAA treated at a single center over 5 years. The most common pathogens were *Salmonella* spp. (74%) and *Streptococcus* spp. (11%). There was a single case of *B. fragilis* who did not survive. 

Matsuyama *et al.* [12] successfully-treated a patient with acute MAA due to *B. fragilis* by placing a dacron graft replacement between the distal arch and the proximal descending aorta with teflon felt reinforcement, and covering the graft with omental flap. 

Tsuji *et al.* [13] successfully treated a patient with a mycotic aneurysm caused by *B. fragilis* of the common iliac artery complicated with vertebral spondylitis. Under midline laparotomy, complete debridement of the infected tissues, in-situ replacement of the common iliac artery with cryopreserved aortic allograft, and iliac bone autotransplantation and omentopexy to fill the debrided cavity were performed. 

Beland *et al.* [14] described a patient suffering from leprosy that developed *B. fragilis* sepsis and MAA.

### *Clostridium septicum* Infections 

Takano *et al.* [15] reported a case of mycotic aneurysm of the infrarenal abdominal aorta infected by *C. septicum* and reviewed 18 addition cases. Of the total of 19 cases of aneurysm caused by *C. septicum* that have been reported, [16-33] at the time of the review the aneurysm was located in the abdominal aorta in 6, the thoracic in 4, the thoracoabdominal in 2, the iliac artery in 2, the popliteal artery in 1, and the thoracic aorta and abdominal aorta (double aneurysm) in 1 patient. In addition, two cases of aortic dissection caused by *C. septicum* infection have been reported [32, 33]. In these 19 cases, there were 14 with colon neoplasm, 11 of which were colon cancer. Ten of the 12 patients who underwent vascular surgery survived, whereas all 7 who did not undergo surgery died, mostly from aneurysmal rupture. Thus, surgical treatment seems to be needed to achieve optimal results. 

A typical finding of clostridial mycotic aneurysms in the CT scan is gas formation surrounding the aorta or peripheral arteries [15]. Clostridia can proliferate in tissues when oxidation-reduction falls or the pH is reduced, which may occur with arterial injury, necrotic tissue, or anoxic tissue with lactic acid accumulation. For this reason, clostridial infection is frequently associated with gastrointestinal or hematologic malignancy. Kornbluth *et al.* [34] reported an associated malignancy in 81% of patients with *C. septicum* infection, and other studies have reported similar findings [17, 35]. It is believed that ulcerative lesions of the gastrointestinal tract, especially colon carcinoma, can allow clostridial organisms to enter the bloodstream and seed an atherosclerotic focus in the aorta, resulting in the development of MAA [36]. The diagnosis of clostridial MAA requires therefore a thorough search for an occult malignancy. 

Three additional cases of MAA due to *C. septicum* not included in the above review were also recently reported [37-39].

*Clostridium paraputrificum* was also reported to be associated in a single case of AMA in a patient with a myeloproliferative disorder and a necrotic carcinoma of the colon [40]. 

### *Propionibacterium acnes* Infections

In addition to the patient with *P. acnes* MAA, described by Brook and Frazier [4], another patient was reported by Galema *et al.* [41].

Data recently reported by Marques da Silva *et al.* [42] sheds light about the potential role of *Propionibacterium* species as well as other anaerobic bacteria in the etiology of aortic aneurysms. The authors evaluated 53 samples from aneurysm walls that were collected from 49 patients during reconstructive surgery. The tissue specimens were sectioned and cultured under anaerobic conditions. Anaerobic cultivation yielded bacteria in 14 of the 53 samples (26.4%). All bacteria were gram-positive cocci or rods from nine genera and 12 species. Five cultures (35%) were mixed, containing two bacterial species. Mixed aerobic and anaerobic species were found in four samples (28.5%). Anaerobic bacteria were recovered from 10 of 14 positive cultures (71%). Among the anaerobes found were *P. acnes* (5 instances), and *Propionibacterium granulosum, Actinomyces viscosus, Actinomyces naeslundii,* and *Eggerthella lenta* (1 each). In further investigation Marques da Silva *et al.* [43] examined 10 aortic aneurysms for the presence of bacterial DNA using polymerase chain reaction (PCR) targeting the 16S ribosomal RNA (rRNA) gene, followed by cloning and sequencing. Sequences of *P. acnes* were identified in five samples and *Prevotella melaninogenica* were detected in one sample. 

The authors concluded that the presence of bacteria in aortic aneurysms did not necessarily imply a causal relationship in aneurysm initiation and development, and some of these bacteria might have been secondary colonizers of a previously damaged vessel. However, their presence demonstrates the ability of anaerobic bacteria to reach the aortic aneurysms and participate in some instances in the mycotic process.

## ANTIMICROBIAL MANAGEMENT OF MYCOTIC ANEURISM DUE TO ANAEROBIC BACTERIA

Management of MAA is one of the most challenging clinical problems. Successful resolution of MAA depends on early diagnosis, prolonged antibiotic therapy, and timely surgery. Management options of MAA include antibiotic therapy alone or in combination with surgical or endovascular therapy. No data is available regarding the effects of medical therapy alone for aneurysm infected by anaerobic bacteria. Therefore this section addresses the antimicrobial choice of agents to be used with or without surgery. Surgical treatment seems to be needed to achieve optimal results based on the review of *C. septicum* MAA by Takano *et al.* [15].

The initial therapy, which is generally empirical should cover the most likely infecting organisms. Antimicrobial effective against anaerobic bacteria should be administered empirically whenever their presence is suspected or proven. The final choice of antimicrobial agents should be based on isolation of specific organisms, aerobes as well as anaerobes from the blood or infected site and antimicrobial susceptibility testing done. Parenteral antimicrobial therapy for four to six weeks is recommended for the treatment of a MAA [44]. A longer treatment is considered when parameters of inflammation such as C-reactive protein, erythrocyte sedimentation rate, and white cell count do not return to normal. 

The susceptibility of anaerobic bacteria to antimicrobial agents has become less predictable and resistance to several antimicrobial agents by *B. fragilis* group and other anaerobic Gram-negative bacilli has increased over the past decade [3, 45]. It is therefore important to perform susceptibility testing to the isolates recovered from patients with MAA. 

The parenteral antimicrobials generally effective against anaerobic bacteria are clindamycin, metronidazole, chloramphenicol, cefoxitin, a combination of penicillin (i.e., ticarcillin, ampicillin) plus a beta-lactamase inhibitor (i.e., clavulanate, sulbactam), tigecycline, moxifloxacin, and the carbapenems (i.e. imipenem, meropenem). Aminoglycosides or a quinolones generally is added to clindamycin, metronidazole and, occasionally, cefoxitin to provide coverage for enteric bacteria, and to a carbapenem to cover for Pseudomonas [3, 45]. Penicillin is added to metronidazole to cover for microaerophilic streptococci, *Actinomyces* spp. and *Propionibacterium* spp. Penicillin is added to clindamycin to supplement its coverage against *Peptostreptococcus* spp. and other Gram-positive anaerobic organisms.

Antimicrobial agents that generally provide coverage for methicillin-susceptible *S. aureus* as well as for anaerobic bacteria include cefoxitin, clindamycin, carbapenem, tigecycline, and combinations of penicillin (e.g., ticarcillin) and a beta-lactamase-resistant penicillin. A glycopeptide (e.g., vancomycin), daptomycin, tigecycline, linezolid, and quinupristin/dalfopristin should be administered in cases in which methicillin-resistant *S. aureus* is present or suspected. 

## CONCLUSIONS

Anaerobic Gram-negative bacilli (mostly *B. fragilis* group), *Clostridium* spp. (mostly *C. septicum*), and *Propionibacterium* spp. (mostly *P. acnes*) are the predominant anaerobes isolated from MAA. Because anaerobic Gram-negative bacilli have increased their resistance to penicillins and other antimicrobials in recent years, complete identification and antimicrobial susceptibility testing are essential for the management of infections caused by anaerobic bacteria. The mainstay of treatment of MAA involving anaerobes includes the use of antibiotic therapy effective these organisms. 
